# Extracellular vimentin is a novel axonal growth facilitator for functional recovery in spinal cord-injured mice

**DOI:** 10.1038/srep28293

**Published:** 2016-06-21

**Authors:** Michiko Shigyo, Chihiro Tohda

**Affiliations:** 1 Division of Neuromedical Science, Institute of Natural Medicine, University of Toyama, 2630 Sugitani, Toyama 930-0194, Japan

## Abstract

Vimentin, an intermediate filament protein, is an intracellular protein that is involved in various cellular processes. Several groups have recently reported that vimentin also appears in the extracellular space and shows novel protein activity. We previously reported that denosomin improved motor dysfunction in mice with a contusive spinal cord injury (SCI). At the injured area, astrocytes expressing and secreting vimentin were specifically increased, and axonal growth occurred in a vimentin-dependent manner in denosomin-treated mice. However, the axonal growth that was induced by extracellular vimentin was only investigated *in vitro* in the previous study. Here, we sought to clarify whether increased extracellular vimentin can promote the axonal extension related to motor improvement after SCI *in vivo*. Extracellular vimentin treatment in SCI mice significantly ameliorated motor dysfunction. In vimentin-treated mice, 5-HT-positive axons increased significantly at the rostral and central areas of the lesion, and the total axonal densities increased in the central and caudal parts of the lesioned area. This finding suggests that increased axonal density may contribute to motor improvement in vimentin-treated mice. Thus, our *in vivo* data indicate that extracellular vimentin may be a novel neurotrophic factor that enhances axonal growth activity and motor function recovery after SCI.

Reconstruction of neuronal networks after trauma in the adult central nervous system (CNS) is still difficult to achieve in both clinical and basic science studies. Spinal cord injury (SCI) causes irreversible damage to the spinal cord and leads to serious motor, sensory and/or autonomic nervous system dysfunction due to disruption of the descending and ascending fiber tracts. Although it has been thought for decades that adult CNS axons do not have the plasticity needed to repair lesions through regeneration, numerous studies have suggested that CNS axons have the ability to regenerate and that the regrowth of injured and/or spared axons may lead to locomotor dysfunction recovery^1^,^2^,^3^. In contrast, several types of inhibitory factors that are derived from glial cells have been shown to potently obstruct axonal restoration^4^,^5^. Several studies have confirmed that glial scarring, which is an inhibitory obstacle, is formed by reactive astrocytes, which generate chondroitin sulfate proteoglycans (CSPGs); these molecules are known to inhibit axonal growth^6^,^7^,^8^,^9^,^10^. Thus, overcoming CSPG inhibition is necessary to achieve functional recovery after SCI.

In a previous study, we developed a novel compound, denosomin, that exhibited axonal growth activity in cultured neurons^11^. Denosomin treatment in SCI mice improved motor function and both extended and enhanced axonal growth (the serotonin (5-HT)-positive tract)^12^. Interestingly, denosomin treatment increased astrocyte proliferation at the injury site and also promoted vimentin release from those astrocytes^12^. Elongated axons following denosomin treatment were well correlated with extracellular vimentin^12^. Prior to that study, the functional role of secreted vimentin was unknown. However, the extracellular addition of recombinant vimentin to cultured cortical neurons resulted in axonal growth that was even sustained on CSPG-coated dishes^12^.

Vimentin is an intermediate filament protein and is generally recognized as an intracellular protein that is involved in cellular processes, such as cell adhesion and cell migration^13^,^14^,^15^,^16^. Although two reports have suggested vimentin secretion from unstimulated astrocytes^17^,^18^, there have been no reports of the potential role of extracellular vimentin in specific signaling to cells. Our previous study demonstrated that extracellular vimentin directly binds and activates insulin-like growth factor 1 receptor (IGF1R) in neurons^19^. The new discovery that extracellular vimentin induces axonal growth in cultured cortical neurons suggests that extracellular vimentin may act as a novel nerve growth factor. Therefore, in this study, we sought to determine whether increased extracellular vimentin could promote axonal growth and locomotor recovery in SCI mice.

## Results

### Extracellular vimentin treatment improved motor dysfunction in contusive SCI mice

To investigate the effect of extracellular vimentin on motor improvement in SCI mice, recombinant vimentin or vehicle solution (ACSF; artificial cerebrospinal fluid) was continuously administered into the lateral ventricle using a micro-osmotic pump for 21 days following SCI.

**Figure 1:**
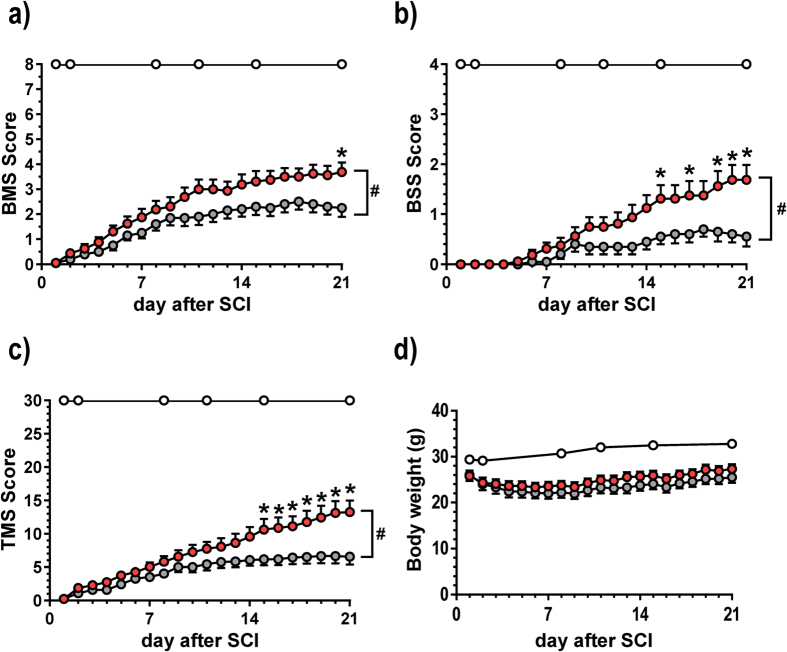
Vimentin enhances the hind limb function in SCI mice. The BMS score (**a**), BSS score (**b**), TMS (**c**) and body weight (**d**) were measured. SCI mice received vimentin (red circles, 8 mice, 16 hind limbs, n = 16) or vehicle solution (grey circles, 10 mice, 20 hindlimbs, n = 20). Sham-operated mice were administered vehicle solution (open circles, 9 mice, 18 hindlimbs, n = 18). ^#^*P* < 0.0001, drug × day interaction analyzed using a repeated-measures two-way ANOVA; *P < 0.05 compared with the vehicle-treated SCI mice on the same day using the Bonferroni *post hoc* test.

**Figure 2:**
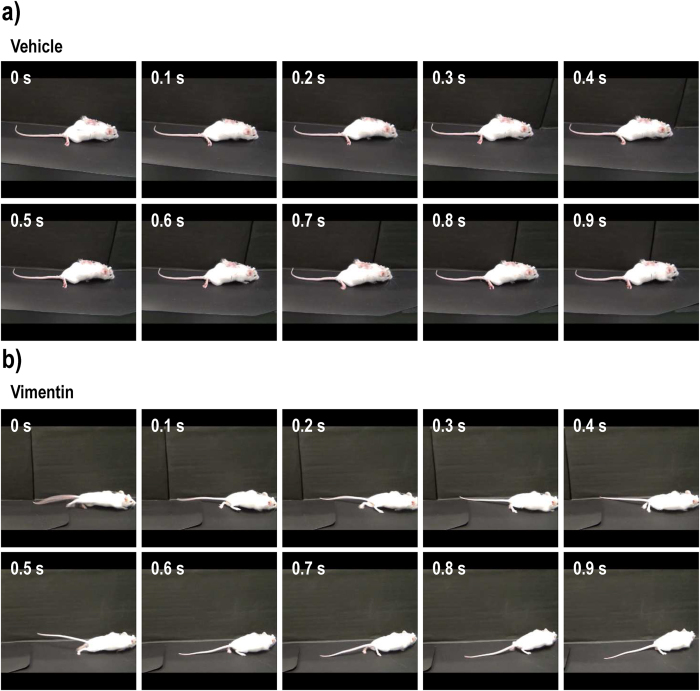
Vimentin enhances the hind limb function in SCI mice. At 21 days after spinal cord injury, the movements of the mice during walking were evaluated. Images that were sequentially captured for 0.9 s are shown in (**a**,**b**).

The motor function in the right and left hindlimbs was scored separately using the Basso Mouse Scale (BMS) (Fig. 1a), Body Support Scale (BSS) (Fig. 1b) and Toyama Mouse Score (TMS) (Fig. 1c). The BMS, BSS and TMS scores of vimentin-treated mice gradually and significantly increased compared with control SCI mice over the 21-day course of administration. The F and *P* values of interaction effects between treatment and days after SCI were calculated as follows: F(2, 680) = 3.18; *P* < 0.0001 in BMS, F(2, 680) = 5.59; *P* < 0.0001 in BSS, and F(2, 680) = 7.25; *P* < 0.0001 in TMS. Sham-operated mice received the maximum scores on all tests. We repeated these experiments twice and confirmed that a similar degree of motor dysfunction was reproducibly observed in our contusion model. Vimentin administration did not cause a loss in body weight (Fig. 1d) or induce abnormal behaviors in the mice (data not shown). At 21 days post-injury, the movements of the mice during walking were captured (Fig. 2). As shown in Fig. 2a, the ACSF-treated control mice dragged their legs during movement. As shown in the 0.9 s of sequentially captured images, the ACSF-treated control mice showed an abnormal walking gait. In contrast, the mice in the vimentin-treated group showed high ankle mobility and trunk stability during walking (Fig. 2b).

### Vimentin treatment increased NF-H-positive axonal densities at the SCI site in SCI mice

**Figure 3:**
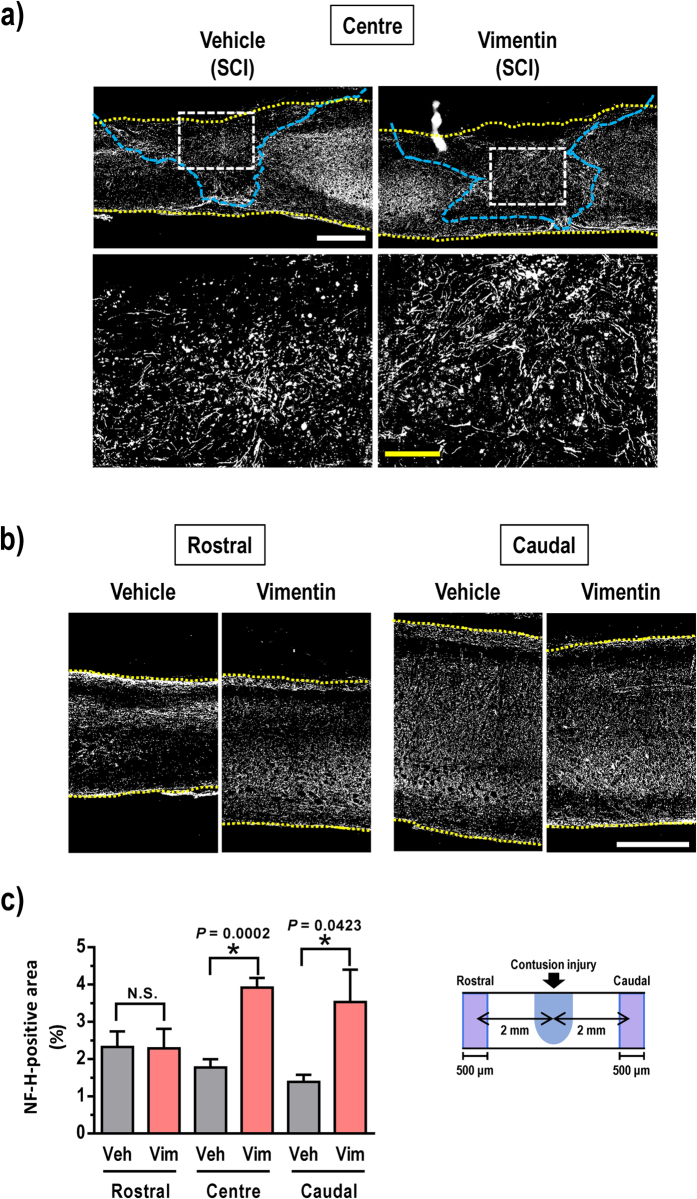
Vimentin enhances the axonal density in SCI mice. Sagittal sections of the spinal cord of vehicle-treated or vimentin-treated SCI mice were immunostained for NF-H (**a**,**b**). The relative areas of immunostaining were measured in three locations of the spinal cord: at the center of the lesion and the sites that were 2 mm rostral and 2 mm caudal to the injury center (**c**). The white dashed squares are magnified in the right panels in (**a**). The yellow dashed lines in (**a**,**b**) indicate the outlines of the spinal cords. The blue dashed area in (**a**) corresponds to the glial scar region. *P < 0.05 vs. vehicle, unpaired *t*-test (two-tailed), one-way ANOVA Bonferroni *post hoc* test. Veh, vehicle; Vim, vimentin. In (**c**), Veh, n = 5; Vim, n = 5. The white scale bar indicates 500 μm, and the yellow bar indicates 100 μm.

The injured spinal cords of vimentin-treated SCI mice were examined by immunohistochemistry. First, positive staining for NF-H (an axonal marker) was assessed at the lesion center (Fig. 3a). The NF-H-positive area was significantly higher in the center of the lesion in the vimentin-treated group than in the vehicle-treated and non-injured control groups (Fig. 3b). NF-H-positive staining was also measured in regions that were rostral and caudal to the lesion center (Fig. 3b). After vimentin treatment, the NF-H-positive axonal staining was significantly increased in the caudal area but not in the rostral area (Fig. 3c). Therefore, these results suggest that extracellular vimentin promotes axonal elongation at the lesion center and the area caudal to the lesion center.

### Vimentin treatment increased the 5-HT-positive axonal densities in the raphespinal tracts in the spinal cord tissues of SCI mice

**Figure 4:**
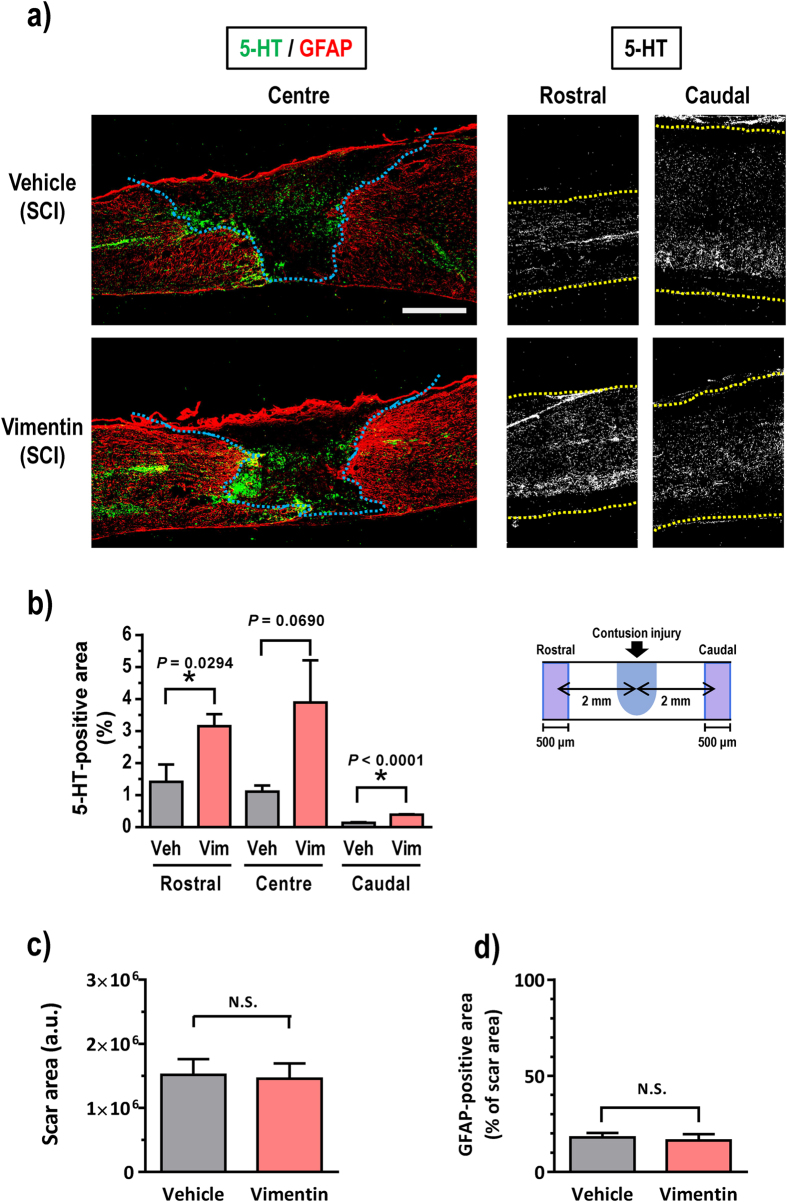
Vimentin enhances the 5-HT-positive axonal density in SCI mice. Sagittal sections of the spinal cord of control, vehicle-treated or vimentin-treated SCI mice were immunostained for 5-HT (**a**). The relative areas of immunostaining were measured in three locations of the spinal cord: at the center of the lesion and at sites that were 2 mm rostral and 2 mm caudal to the lesion center (**b**). The blue dashed lines in (**a**) indicate the outlines of the glial scar. The yellow dashed lines in (**a**) indicate the outlines of the spinal cord. The size of scar area was measured (**c**). The area of GFAP-positive reactive astrocytes was measured (**d**). *P < 0.05 vs. vehicle, unpaired *t*-test (two-tailed), one-way ANOVA Bonferroni *post hoc* test. Veh, vehicle; Vim, vimentin; In (**b**), Veh, n = 5; Vim, n = 5. In (**c**,**d**), Vehicle, n = 10; Vimentin, n = 6. The scale bar indicates 500 μm.

Next, spinal cord sections were immunostained for 5-HT to visualize raphespinal tracts in the spinal cord because these tracts are known to predominantly regulate locomotor function^19^,^20^,^21^. Positive 5-HT staining was detected in the lesion center and in regions 2 mm rostral and caudal to the lesion center (Fig. 4a). Many more 5-HT-positive axons were detected along the scar rim and in the rostral area in the vimentin-treated mice than in the vehicle-treated group. The quantitative evaluation of 5-HT-positive fiber-like staining is shown in Fig. 4b. Compared with the vehicle-treated group, the vimentin-treated animals had a significantly larger 5-HT-positive area at sites that were 2 mm rostral and caudal to the lesion center. At the lesion center, the 5-HT staining increased non-significantly after vimentin treatment. Compared with the 5-HT staining in the non-injured control group, the 5-HT-positive areas in the vimentin-treated group were larger at the lesion center and rostral to the lesion center (Fig. 4b). 5-HT-positive axons rarely crossed over the glial scar in either group; however, at the rostral site, the vimentin-treated mice showed slightly more 5-HT-positive axons that extended to the caudal region (Fig. 4b). The scar area was defined as the inside space that was surrounded by GFAP-positive astrocytes. The scar sizes of the analyzed areas did not differ between groups (Fig. 4c). In addition, the area of GFAP-positive reactive astrocytes was not different between groups (Fig. 4d). These results indicate that extracellular vimentin enhances the elongation of 5-HT-positive fiber tracts.

## Discussion

We previously demonstrated that denosomin increased the number of vimentin-expressing astrocytes inside the glial scars of SCI mice and that 5-HT-positive axonal growth occurred in a vimentin-dependent manner^12^. Here, we showed that extracellular vimentin promoted axonal growth and also ameliorated motor dysfunction in SCI mice. This is the first report of the effects of extracellular vimentin in an *in vivo* SCI model.

Spinal motor neurons terminate in the skeletal muscle in hindlimbs and are regulated by multiple descending tracts, propriospinal neurons and interneurons^20^,^21^. The serotonergic raphespinal tract is one of the main descending tracts that regulate voluntary movement^22^,^23^,^24^. Extracellular vimentin administration in the SCI mice enhanced the growth of 5-HT-positive axons in the areas that were rostral and caudal to the lesion (Fig. 4). Furthermore, at the lesion center, the number of 5-HT-positive axons increased following vimentin treatment. NF-H-positive axons were notably increased both at the lesion center and at the caudal area following vimentin treatment (Fig. 3). Because NF-H-positive staining should appear in a majority of axons, the increase in axons at the center and caudal areas may reflect the elongation from a variety of sources, including interneurons, propriospinal neurons, and/or descending tracts, such as the raphespinal, corticospinal or rubrospinal tract. The regeneration of axons descending from the brain and propriospinal relay connections are important for functional recovery after SCI^25^,^26^. In the mammalian spinal cord, the locomotor circuit is functionally organized by interneurons^27^. Because projections to motor neurons by the serotonergic raphespinal tracts are established by either direct or interneuron-mediated indirect pathways, it is possible that some of the observed elongated 5-HT-positive axons may innervate motor neurons via interneurons in the spinal cord of extracellular vimentin-treated mice. We supposed that the extracellular administration of vimentin in the cerebral ventricle might affect at multiple levels of the spinal cord and brain areas. IGF1R was shown to be a receptor of extracellular vimentin^19^, and it is expressed widely throughout the brain and spinal cord^28^. Therefore, vimentin most likely stimulates axonal growth via IGF1R through a variety of mechanisms.

Our previous reports have shown that extracellular vimentin induced axonal growth in primary cultured neurons and that this growth even persisted when the neurons were grown on CSPG substrates^12^. Additionally, we showed that denosomin-treated astrocytes secreted vimentin into the extracellular space and that 5-HT-positive axons were especially increased in vimentin-positive areas. These previously published results suggest that extracellular vimentin could potentially contribute to axonal growth in injured areas. Other groups have reported the spontaneous secretion of vimentin from astrocytes without stimulation^17^,^18^. Furthermore, in our previous study, cultured astrocytes released modest levels of vimentin without stimulation. In contrast, denosomin treatment resulted in a four-fold increase in vimentin secretion^12^. The vimentin level is known to increase after contusive injury in the spinal cord^4^. These results may suggest that the extracellular vimentin that is secreted from astrocytes is also related to the spontaneous partial improvement of SCI. However, the exogenous administration of extracellular vimentin may be required for significant recovery.

Our preliminary data show that a single direct microinjection of vimentin to the SCI site was effective in improving motor activity in SCI mice. In the current study, the continuous i.c.v. delivery of vimentin was performed using a micro-osmotic pump, and the vimentin-treated group showed a significant increase in axonal density at the SCI site. This result suggests that an increase in extracellular vimentin contributes to the enhancement of axonal densities in injured spinal cord tissues.

As a mechanism of vimentin-induced axonal growth, we have already demonstrated that IGF1R is the direct target of extracellular vimentin in cultured neurons^19^. IGF1R is broadly expressed in several types of cells in the CNS^29^,^30^. The IGF1R knockout mouse shows abnormal morphology and failure of some tissues and organs during various developmental stages^31^, indicating that IGF1R has an important role in cell growth and/or development. In preliminary experiments, an IGF1R inhibitor, IGF1-analog, showed lethality when administered by i.c.v. injection (data not shown). Therefore, to investigate whether the vimentin-induced effect is mediated by IGF1R *in vivo*, IGF1R conditional knockout mice should be used^32^.

Because neurotrophic factors are important for activating axonal growth^33^, the vimentin-mediated release of neurotrophic factors might be involved in the axonal growth that is induced by vimentin injection. However, our previous *in vitro* data showed that vimentin did not increase the neuronal secretion of IGF1, IGF2 and insulin, which are physiological ligands for IGF1R^19^. Additionally, our unpublished *in vitro* data showed that vimentin-treated astrocytes and vimentin-treated microglia do not secrete axonal growth facilitators. This finding suggests that the vimentin-induced axonal growth and motor function improvements that were observed in SCI mice might not be mediated by other neuron-, astrocyte- or microglia-derived neurotrophic factors. Whether extracellular vimentin influences additional cell types in SCI mice requires further investigation.

Many studies have shown a relationship between vimentin and neurons, but most studies have focused on the role of vimentin as an intracellular protein. One report showed that the expression of vimentin in neuroblastoma cells increased the number of axonal neurites^34^. During development, the corticospinal tract elongates in proximity to vimentin-positive areas^35^. Additionally, Hsu *et al*. showed that vimentin-positive astrocytes induced axonal regrowth after spinal cord hemisection and Schwann cell implantation in adult rats^36^. Another group also suggested that increased vimentin expression is involved in spontaneous recovery after contusive SCI^4^. Together, these reports indicate that vimentin increases axon growth, although these studies focused on its role as an intracellular molecule rather than an extracellular one. In contrast, vimentin secretion was detected in not only astrocytes^17^,^18^ but also activated macrophages^37^ and vascular endothelial cells^38^. Vimentin is categorized as a ‘moonlighting’ or ‘gene-sharing’ protein that has been increasingly found in unexpected cellular locations^39^,^40^. Some of these proteins are derived from a single gene but adopt different properties and functional roles. Vimentin is now recognized as one of these multi-functional proteins, although the differences between its intracellular and extracellular functions and its secretory pathway remain unclear^41^. Extracellular vimentin facilitates axonal growth and increases the number of reactive astrocytes in damaged CNS tissue areas. If the vimentin-secreting function is enhanced in astrocytes, vimentin may contribute to axonal elongation after injury. Further elucidation of the detailed mechanisms underlying the function of vimentin will be essential for developing vimentin-based drug and/or gene therapies.

Our findings suggest that extracellular vimentin facilitates axonal growth and improves motor dysfunction in SCI mice. These data provide unprecedented scientific evidence about vimentin and suggest that it may be a potential therapeutic target for promoting axonal growth.

## Materials and Methods

All experiments were performed in accordance with the Guidelines for the Care and Use of Laboratory Animals of the University of Toyama and the National Institutes of Health Guidelines for the Care and Use of Laboratory Animals. The Committee for Animal Care and Use of the University of Toyama approved the study protocols. All efforts were made to minimize the number of animals used.

### Animals and SCI model experiments

Six- to seven-week-old female ddY mice (SLC, Shizuoka, Japan) were used for the SCI experiments. The mice were housed with ad libitum access to food and water and were maintained under constant environmental conditions (22 ± 2 °C, 50 ± 5% humidity and a 12-hr light: 12-hr dark cycle starting at 07:00). The surgical operations for producing contusive SCI were performed as previously described^12^ with slight modifications. The mice were anesthetized with trichloroacetaldehyde monophosphate (500 mg/kg, i.p.). After laminectomy at the T10 level, contusion injuries were produced by dropping a 6.5 g weight from a height of 3 cm onto the exposed L1 level using a stereotaxic instrument (Narishige, Tokyo, Japan). The incision was closed with sutures.

After SCI surgery, surgical procedures to place the cannula and micro-osmotic pump were performed. The mice were placed in a stereotaxic apparatus to keep the head in a fixed position. The scalp was shaved, followed by a sagittal midline incision to expose the skull. A cannula (Brain Infusion Kit 3, Alzet, Cupertino, CA, USA) was positioned into a lateral ventricle at the following coordinates: bregma −0.22 mm, lateral to the left +1 mm and −2.5 mm depth. The free end of the cannula was connected to a micro-osmotic pump (Alzet model 1004) via a 3.5-cm piece of polyvinylchloride (PVC) tubing (Alzet). The pump was placed into a subcutaneous pocket on the back of the mouse. The cannula was fixed to the skull with Loctite 454 cyanoacrylate. The infusion rate of the micro-osmotic pump was 0.11 μl/hr. The filled pumps were incubated in 0.9% sterile saline at 37 °C for at least 16 hr in a CO_2_ incubator before implantation. For the vehicle-treated group, the micro-osmotic pump and connected PNC tube were filled with artificial cerebrospinal fluid (ACSF) containing 130 mM NaCl, 24 mM NaHCO_3_, 3.5 mM KCl, 1.3 mM NaH_2_PO_4_, 2 mM CaCl_2_, 2 mM MgCl_2_ · 6 H_2_O and 10 mM glucose at pH 7.4. For the vimentin-treated group, the micro-osmotic pump and tube were filled with 1 μg of recombinant human vimentin (ProSpec, Rehovot, Israel) that was dissolved in ACSF. The delivery rate of the solution from the pump was 0.11 μl/hr. In this way, the concentration of vimentin that was delivered to the CSF was always 61 ng/ml. Previously, our study showed that the effective concentration of extracellular vimentin for axonal growth was 10–200 ng/ml^19^. During and after surgery, the mice were placed on a heating pad to maintain body temperature.

### Behavioral evaluation

For behavioral scoring after surgery, the mice were individually placed in an open field (42 cm × 48 cm × 15 cm) and observed for 5 min once per day for a total of 21 days. Open-field locomotion was evaluated using three scoring methods: the 0–8 scale BMS score (without the tail score), which evaluates ankle movement and walking stability^42^; the 0–4 scale BSS score, whose criteria we developed to assess the degree of hindlimb muscle strength for support of the body trunk^12^; and the 0–30 scale TMS, the new criteria that we developed by combining and modifying the BMS and BSS scores^43^. Animals were allowed to move freely in the plastic box. Movements of the left and right hindlimbs were evaluated independently.

### Antibodies

The primary antibodies for immunohistochemistry included a rabbit anti-neurofilament-H (NF-H) polyclonal antibody (dilution 1:1000; Chemicon, Temecula, CA, USA), a mouse anti-GFAP monoclonal antibody (clone G-A-5, dilution 1:1000; Sigma, St. Louis, MO, USA) and a rabbit anti-5-HT polyclonal antibody (dilution 1:3000; Sigma). The secondary antibodies that were used included Alexa Fluor 594-conjugated goat anti-mouse IgG1 (dilution 1:400) and Alexa Fluor 488-conjugated goat anti-rabbit IgG (dilution 1:400; Invitrogen, Carlsbad, CA, USA).

### Immunohistochemistry

After behavioral scoring, the mice were deeply anesthetized and fixed by transcardial perfusion with 4% paraformaldehyde in phosphate-buffered saline. Serial spinal cord slices were obtained as 14-μm sagittal sections using a cryostat (CM3050S, Leica, Heidelberg, Germany). The slices were fixed with 4% paraformaldehyde and stained with antibodies to detect NF-H- or 5-HT-positive axons or GFAP-positive astrocytes. The fluorescence images were captured at a 670 × 890 μm size with a 10 × NA 0.45 dry objective lens (Plan-Apochromat, Carl Zeiss, Oberkochen, Germany) and CCD camera (AxioCam MRm) on an inverted microscope (Axio Observer Z1, Carl Zeiss). The captured images were converted and tiled using the AxioVision Z-stack and Mosaix software system (AxioVision 4.8, Carl Zeiss). The optical densities of the 5-HT- and NF-H-positive axons were determined using ImageJ software (NIH). The center of each lesion was determined based on the pattern of GFAP staining. Fluorescence images were then also captured at 2 mm rostral and 2 mm caudal to the injury center.

### Statistical analysis

Statistical comparisons were performed using repeated-measures two-way ANOVA followed by the Bonferroni *post hoc* test, a one-way ANOVA followed by the Bonferroni *post hoc* test or an unpaired (two-tailed) *t*-test. GraphPad Prism 5 (GraphPad) was used for the statistical analyses. *P*-values less than 0.05 were considered significant. The data are presented as the mean ± SEM.

## Additional Information

**How to cite this article**: Shigyo, M. and Tohda, C. Extracellular vimentin is a novel axonal growth facilitator for functional recovery in spinal cord-injured mice. *Sci. Rep.* **6**, 28293; doi: 10.1038/srep28293 (2016).
